# Hyper Immunoglobulin D syndrome (HIDS): understanding what it is like to live with this rare condition

**DOI:** 10.1186/1546-0096-13-S1-P24

**Published:** 2015-09-28

**Authors:** P Dandekar, J Gregson, R Campbell, F Bourhis

**Affiliations:** 1 Novartis Pharma AG, Basel, Switzerland; 2 MAPI, Uxbridge, UK; 3 MAPI, Nanterre, France

## Introduction

Hyper Immunoglobulin D syndrome (HIDS) is a genetic disorder characterized by recurrent attacks of fever and inflammatory symptoms and is most common in those of French or Dutch descent.[^1^,^2^]

## Objectives

To understand the impact of HIDS on the lives of patients/caregivers, to describe patient's journey from first symptoms and to learn what patients hope to emerge in terms of future therapy, support and information.

## Patients and methods

Fifteen patients with HIDS were recruited across US, Europe and Australia. They completed a 20 page pre-interview questionnaire and an in-depth 90 minute in-home interview. Patient responses were recorded and summarized; for patients with complete data available (n=13), responses were quantified. The topics covered in the interview were symptoms, patient journey, and unmet needs.

## Results

Patients reported periods of wellness and symptomatic flares, where flares were characterised by high fevers and nausea (especially in children), as well as pain. In many cases flares were so severe that patients were bedridden. In children, flares led to severely disrupted education. In caregivers and adult patients, attacks and medical appointments resulted in missed work and limited career choices, leading to financial dependency. HIDS also had an impact on patients’ relationships and social lives by limiting their activities. Severity of symptoms, the duration and frequency of flares decreased with age and treatment. Patients frequently experienced a delay in diagnosis (15 mos-20 yrs), where they were subjected to a variety of diagnostic tests for other conditions. While most patients were initially treated with non-steroidal anti-inflammatories, colchicine, or steroids; they transitioned to biological treatment. Responses to biologic agents varied, although many reported shorter attack duration and frequency, all respondents continued to experience attacks. Around half of patients have switched biologics due to lack of efficacy. Patients and parents seek out information independently at diagnosis, usually via the internet. Patients identified improved treatment efficacy and reduced side effects as the areas in greatest unmet need.

## Conclusions

HIDS significantly impacts the physical, social, emotional and practical/financial aspects of patients’ and caregivers’ lives. Greater awareness of HIDS among HCPs may improve diagnostic delays. Patients expressed interests in gaining a greater understanding of their disease and treatment options. There is an unmet need for therapy that prevents or reduces the number of attacks and which has patient-friendly administration.
